# Experiential learnings from the Nipah virus outbreaks in Kerala towards containment of infectious public health emergencies in India

**DOI:** 10.1017/S0950268820000825

**Published:** 2020-04-23

**Authors:** Rima R. Sahay, Pragya D. Yadav, Nivedita Gupta, Anita M. Shete, Chandni Radhakrishnan, Ganesh Mohan, Nikhilesh Menon, Tarun Bhatnagar, Krishnasastry Suma, Abhijeet V. Kadam, P. T. Ullas, B. Anu Kumar, A. P. Sugunan, V. K. Sreekala, Rajan Khobragade, Raman R. Gangakhedkar, Devendra T. Mourya

**Affiliations:** 1 ICMR-National Institute of Virology, Microbial Containment Complex, Sus-Pashan Road, Pune, Maharashtra, India; 2 Indian Council of Medical Research, Ansari Nagar, New Delhi, India; 3 Government Medical College, Kozhikode, Kerala, India; 4 Government Medical College, Kalamassery, Ernakulam, Kerala, India; 5 Public Health Department, District Medical Officer (Health), Kerala, India; 6 ICMR – National Institute of Epidemiology, Chennai, Tamil Nadu, India; 7 T. D. Medical College, Alappuzha, Kerala, India; 8 ICMR-National AIDS Research Institute, Pune, Maharashtra, India; 9 Kerala unit, ICMR-National Institute of Virology, Alappuzha, Kerala, India; 10 Principal Secretary, Health & Family Welfare, Kerala, India

**Keywords:** Biosafety, containment, Kerala, Nipah virus, public health emerging infection

## Abstract

Nipah virus (NiV) outbreak occurred in Kozhikode district, Kerala, India in 2018 with a case fatality rate of 91% (21/23). In 2019, a single case with full recovery occurred in Ernakulam district. We described the response and control measures by the Indian Council of Medical Research and Kerala State Government for the 2019 NiV outbreak. The establishment of Point of Care assays and monoclonal antibodies administration facility for early diagnosis, response and treatment, intensified contact tracing activities, bio-risk management and hospital infection control training of healthcare workers contributed to effective control and containment of NiV outbreak in Ernakulam.

## Introduction

Nipah virus (NiV), a highly pathogenic *Paramyxovirus*, is one of the ten priority pathogens by World Health Organization Research and Development blueprint 2018 [1]. In India, NiV had caused outbreaks in Siliguri and Nadia districts, West Bengal state with case fatality rate (CFR) of 68% and 100%, respectively [2–4]. The country witnessed NiV outbreak in Kozhikode district, Kerala in 2018 with CFR 91% [5]. This outbreak raised an alarm for strengthening the public health system for an effective response to such emergencies. Consequently, the Indian Council of Medical Research (ICMR) undertook initiatives on public health preparedness and research on NiV as a priority along with the national and international stakeholder's liaisons.

We describe herewith the public health response for NiV disease containment in Kerala, the challenges faced and the efforts made to prevent/control the NiV secondary transmission thereby filling the critical gaps in healthcare settings.

### Confirmation of NiV infection in the suspected case and circulating genotype

A 21-year-old male resident of Ernakulam district, Kerala (engineering student at a college in Thodupuzha, Idukki district, Kerala with a 10-day travel history at Thrissur, Kerala) was admitted to a private hospital in Ernakulam on 30 May 2019. He had a 7-day history of high-grade intermittent fever, headache which later involved into encephalitis with features of irritability, gait imbalance and altered sensorium. Magnetic resonance imaging scan indicated multiple small infarcts in the cerebellum, medulla oblongata and pons. Suspecting NiV infection, throat swab, cerebrospinal fluid (CSF), blood and urine samples were sent to the ICMR-National Institute of Virology (NIV), Pune for confirmation on 3 June 2019.

The urine sample was tested positive for NiV by the point of care (PoC) (*developed and standardised by ICMR-NIV, Pune*) and real-time reverse transcriptase-polymerase chain reaction (qRTPCR) [6]. The entire NiV genome (17 Kb) was retrieved by Next Generation Sequencing [7]. The NiV sequence detected was 99.70% identical to the sequence retrieved from the 2018 Kerala human samples (Accession number: MH523641) and belonged to the Bangladesh genotype. The serum sample also tested positive for anti-Nipah human Immunoglobulin M (IgM) and anti-Nipah human IgG antibodies by Enzyme-Linked Immunosorbent Assay (ELISA) using reagents provided by the Centre for Disease Control and Prevention (CDC), Atlanta, USA. On 4 June 2019, the Ministry of Health and Family Welfare, Government of India, declared a NiV outbreak.

### ICMR response to Ernakulum, Kerala NiV outbreak [2019]

ICMR constituted a multidisciplinary team including experts from ICMR-National Institute of Epidemiology, Chennai; ICMR-NIV, Pune; ICMR-National AIDS Research Institute, Pune; Government Medical College (GMC), Kozhikode and Government TD Medical College, Alappuzha. The team's responsibilities included establishing and coordinating facilities for prompt laboratory diagnosis of suspected patients; clinical examination and identification of cases; epidemiological studies and contact tracing; investigating the NiV presence in bats; and monoclonal antibodies (m102.4) preparations and administrations in Nipah cases, if necessary.

### Guidelines for risk categorisation during contact tracing

The team provided guidelines for the risk categorisation algorithm during the contact tracing activities conducted by the district surveillance teams. A total of 330 contacts (family members, neighbours, friends, and healthcare workers (HCWs)) were traced and categorised into low and high risk. The high-risk category included individuals with either a history of direct contact with body fluids (blood, urine, saliva, etc.) of the confirmed NiV case/a probable case that died without laboratory confirmation or having spent about 12 h nearby or in closed space with confirmed NiV case. A total of 52 contacts of the index case (35 hospital staff, 11 community members, and 6 family members) were categorised into the high-risk category. Rest were low-risk contacts defined as those having contact with the confirmed NiV case through touching or contact with clothes, linen, or any other items. The case definitions for NiV surveillance of suspect/probable/confirmed cases were also reviewed and updated.

### Establishment of on-site NiV diagnostic facility at GMC, Ernakulam, Kerala

Immediately, the state government set up an isolation facility at GMC, Ernakulam for observation and management of suspected cases until their NiV confirmation. Following the biosafety guidelines and standard operating procedures (SOPs) based on the experience of the 2018 NiV outbreak, the team set up an on-site field laboratory within 1 day for diagnostics including POC, qRT-PCR and ELISA. A total of 57 suspected cases were tested for NiV infection and were found negative. Prompt and quick testing at this laboratory played a major role in reducing panic and greatly facilitated patient care as well as prevention and control strategies.

During the 2018 NiV outbreak in Kozhikode, an experimental therapeutic m102.4 was imported from Queensland, Australia for treatment of NiV infected patients on compassionate ground. A medical board was constituted, including a pharmacologist from the ICMR team to guide decisions for the management of NiV case-patient(s). The guidelines and consent form for the administration of m102.4 were prepared.

### Pteropus bat survey to track the source of NiV infection

*Pteropus medius* bats (*n* = 141) were captured. Throat and rectal swabs were collected from five roosting sites (Thodupuzha and Muttam in Idukki district and Aluva, Thuruthipuram and Vavakad areas in Ernakulam district) as per travel history details of Nipah case. Four samples tested positive for NiV RNA [6, 8]. Anti-NiV bat IgG antibodies were detected in 12/58 serum samples of tested *Pteropus* bats.

### Challenges faced in healthcare settings

#### Isolation facility

Precautions for standard droplets and contact with the patient were major concerns for NiV containment. Poor triage techniques, poor understanding of the concept of rapid isolation, overcrowding in the out-patient and in-patient department and lack of isolation facility increased chances of the nosocomial spread. No structured protocol was readily available with regards to transferring patients into isolation facilities. Among hospital infection control practices, surface disinfection and regular cleaning of the isolation ward were practised to reduce the risk of secondary-transmission. Protocols for passive surveillance of cases, fever triage, admission procedures, and restriction of visitor access including limited movement were emphasised to minimise potential exposures.

#### Personal protective equipment

Personal protective equipment (PPE) required for NiV containment were the coveralls, N95 masks, shoe cover, safety goggles and/or face shield. However, lack of availability of good quality PPEs and inadequate training of HCWs for donning and doffing became a challenge. We identified a lack of emphasis on biosafety issues related to donning and doffing in the training programs of HCWs. Lack of trained staff leads to overburdening of the few trained staff with psychological impact due to fear of exposure and death from the deadly pathogen. Lack of concept of an observer at each donning and doffing step and unavailability of hand washing stations at the doffing areas, with poor hand hygiene techniques increases the challenge.

#### Bio-medical waste management

All the medical equipment, sharps, linens and used healthcare products such as soiled absorbent pads or dressings, disposable kidney-shaped emesis pans, portable toilets pans, used PPEs were disinfected using 2–5% Lysol/5–10% freshly prepared household bleach and then sent for autoclave or incineration. NiV exposure is fatal; hence, training becomes an important part and the criteria of elimination of the usage of sharps and unwanted pricks to the patient are to be strictly avoided.

#### Handling of human remains

Secretions from infected dead bodies pose high-risk for the transmission; hence, extreme care was taken while handling the dead bodies. The availability of air-impermeable body bags was a challenge. The human remains should never be handed over to the relatives. Training of the mortuary staff and the medical officer were important while handling the human remains including the disinfection of the human remains and sealing in body bags. Besides, hospital/district authorities were guided for taking care of cremation either by electric method (preferably) or deep burial, as per the religious background of the patient. Dedicated vehicles/ambulances were defined with trained staff to carry the human remains and disinfected after usage.

#### Decontamination of the ambulance

A protocol was designed for a trained three-person team, in which two people donned in PPE can work in a hot zone for the disinfection of ambulance. This third-person donned in PPE in the cold zone (5–10 feet away) will document the decontamination process and can be utilised for other assistance if needed. In the limited resource hospital setting, finding an appropriate site for ambulance disinfection itself was a big challenge. It was emphasised to disinfect the ambulance each time it carried NiV suspected, probable, confirmed or human remains.

#### Terminal decontamination of NiV treatment centre

Terminal decontamination becomes an important challenge after the outbreak ends as no defined standards and guidelines are currently available. Hospital infection control in-charge was given the responsibility for the inspection of the facility before and after terminal cleaning and decontamination. It was ensured that the plan for terminal decontamination was understood by all the staff members, who were competent in cleaning and disinfection. Visual inspection of all the surfaces for a sign of wear and tear, decay or overall disrepair (e.g., mattresses, furniture and equipment) were identified for safe disposal/incineration including; all non-intact objects/equipment. Surfaces that were intact and could withstand rigorous cleanings like stainless steel furniture or beddings underwent cleaning, disinfection and surface decontamination using 2–5% Lysol/5–10% freshly prepared household bleach. The cleaning was followed by the fumigation of the facility with the desired concentration of potassium permanganate and formaldehyde solution depending upon the cubic feet area of the facility. The facility was opened for reuse until the safe completion of the process was done and documented by the hospital infection control authorities and district/state health officials.

The team conducted intensive training sessions on risk mitigation, donning and doffing of PPE to local medical practitioners, nursing staff and the public health team. Later, faculty and laboratory technologists of GMC, Ernakulam were also trained on screening Dengue, Japanese encephalitis and West Nile virus infection for all NiV negative cases [9, 10]. About 30 000 HCWs of different cadres were trained within 2 weeks of the outbreak onset.

## Conclusion

Preparedness is the key element in response to the containment of highly infectious disease outbreaks. The trained and experienced team-work could achieve contact tracing, proper risk communication and establishment of an on-site laboratory, the monoclonal antibody administration facility along with quick identification of the NiV source in *Pteropus* bats. All these efforts put together could help in the early containment of Nipah disease in 2019.
